# Short-term spinal cord stimulation is an effective therapeutic approach for herpetic-related neuralgia—A Chinese nationwide expert consensus

**DOI:** 10.3389/fnagi.2022.939432

**Published:** 2022-09-20

**Authors:** Wuping Sun, Yi Jin, Hongjun Liu, Dong Yang, Tao Sun, Yaping Wang, Yinghui Fan, Xiaochong Fan, Xiaohong Jin, Li Wan, Ke Gu, Zhiying Feng, Yiming Liu, Peng Mao, Tao Song, Wang Dequan, Donglin Xiong, Guoming Luan, Xiaoping Wang, Bifa Fan, Lizu Xiao

**Affiliations:** ^1^Department of Pain Medicine and Shenzhen Municipal Key Laboratory for Pain Medicine, Shenzhen Nanshan People's Hospital and the 6th Affiliated Hospital of Shenzhen University Health Science Center, Shenzhen, China; ^2^Department of Pain Management, Jinling Hospital, Nanjing, China; ^3^Department of Pain Medicine, Institute of Anesthesiology and Critical Care Medicine, Union Hospital, Tongji Medical College, Huazhong University of Science and Technology, Wuhan, China; ^4^Department of Pain Management, Shandong Provincial Hospital Affiliated to Shandong First Medical University, Jinan, China; ^5^Department of Pain Management and Anesthesiology, The Second Xiangya Hospital, Central South University, Changsha, China; ^6^Department of Pain Management and Anesthesiology, Renji Hospital, School of Medicine, Shanghai Jiaotong University, Shanghai, China; ^7^Department of Pain Medicine, The First Affiliated Hospital of Zhengzhou University, Zhengzhou, China; ^8^Department of Pain Management, The First Affiliated Hospital of Soochow University, Suzhou, China; ^9^Department of Pain Management, The State Key Clinical Specialty in Pain Medicine, The Second Affiliated Hospital, Guangzhou Medical University, Guangzhou, China; ^10^Department of Pain Management, Sanbo Brain Hospital Capital Medical University, Beijing, China; ^11^The First Affiliated Hospital, Zhejiang University School of Medicine, Hangzhou, China; ^12^Department of Pain Medicine, Peking University People's Hospital, Beijing, China; ^13^Department of Pain Medicine, China-Japan Friendship Hospital, Beijing, China; ^14^Department of Pain Medicine, The First Hospital, China Medical University, Shenyang, China; ^15^People's Hospital of Xinjiang Uyghur Autonomous Region, Ürümqi, China; ^16^Department of Neurosurgery, Comprehensive Epilepsy Center, Sanbo Brain Hospital Capital Medical University, Beijing, China; ^17^Department of Pain Management, The First Affiliated Hospital, Jinan University, Guangzhou, China

**Keywords:** herpetic-related neuralgia (HN), short term spinal cord stimulation (st-SCS), acute herpetic neuralgia (AHN), subacute herpetic neuralgia (SHN), postherpetic neuralgia (PHN)

## Abstract

**Purpose:**

Short-term spinal cord stimulation (st-SCS) has been widely used to treat herpetic-related neuralgia (HN) in China for several years, but is still heavily debated as it has no strong evidence in clinical application. Therefore, a questionnaire survey among the Chinese pain specialist workgroup of the Chinese Neuromodulation Society and Chinese Medical Doctor Association was carried out to achieve a consensus about the clinical use of st-SCS for HN treatment.

**Methods:**

The contents of the questionnaire include basic information about doctors (hospital level, work experience, training, procedure numbers, etc.), efficacy, indications, and contraindications of st-SCS, operation conditions, and preoperative preparation of st-SCS, and the prospect of the st-SCS procedure. Initially, the survey was conducted on 110 experts who have practiced the st-SCS procedure from all over the provinces in China. Finally, valuable data was calculated from the 110 questionnaires excluding the doctors with <1 year of experience of st-SCS, <10 cases of procedures per year, and no standard training in SCS technique.

**Results:**

Based on the 110 questionnaires, it is estimated that 5,000 to 10,000 cases of electrical stimulation are carried out nationwide each year. Sixty-nine valid questionnaires acquired from senior pain physicians were more valuable and specialized in the efficacy, indications, and contraindications of st-SCS for HN. It was commonly agreed (97.10%) that the HN patients with <3 months will obtain good effectiveness (patient satisfaction rate ≥50%). Almost all (98.55%) agreed that st-SCS can be used in SHN patients, there was a common agreement (72.46%) that AHN patients are an indication of st-SCS, and more than half agreement (53.62%) that st-SCS may be fit for early PHN (3–6 months). A common agreement (79.71%) was achieved that more than half of HN patients had the experience of nerve block or nerve pulsed RF. A similarly large number of experts 57/69 (82.61%) agreed that an 80% paresthesia coverage should be achieved at the test stimulation and 57/69 (82.61%) agreed that the treatment of st-SCS need be persistent for 1–2 weeks.

**Conclusions:**

Early HN patients can get an effective outcome from the treatment of st-SCS and maybe the indication of st-SCS. Moreover, standardized training for pain physicians and basic research and clinical studies are warranted.

## Introduction

Herpes zoster (HZ) is a neurocutaneous viral disease, one of the most common acute skin conditions, that may occur at any age. Even if the rash eventually resolves, patients may experience persistent and severe pain as a consequence. Herpetic-related neuralgia (HN) is the most common complication in elderly patients with herpes zoster and it can be classified as acute herpetic neuralgia (AHN) within 1 month of the onset, subacute herpetic neuralgia (SHN) within 3 months of onset, and postherpetic neuralgia (PHN) after 3 months (Arani et al., 2001; Dworkin et al., 2008; Johnson and Rice, 2014). PHN incidence is generally reported from 10 to 35% among HZ patients, with higher rates correlating to elderly and immune-compromised populations (Kawai et al., 2014). Patients younger than 50 years are with a 2% risk of suffering PHN and it increases to 20% over the age of 50 years. A 10-year increase in age in the range of 50–79 years is associated with a 70% increased risk of PHN (Helgason et al., 2000; Johnson and McElhaney, 2009; Kawai et al., 2014; Forbes et al., 2016; Li et al., 2016). It has been estimated that the prevalence of HZ in China is 7.7%, and 29.8% of which develop PHN subsequently (Yang et al., 2019). Given the chronic nature of PHN, it is often associated with depression, anxiety, poor sleep quality, and declined physical activity, which together could significantly affect the individual's quality of life (Drolet et al., 2010; Mizukami et al., 2018).

To date, it's still challenging to achieve optimal symptomatic relief for patients with HN. Although several pharmacological agents, such as anticonvulsants (e.g., gabapentin, pregabalin), tricyclic antidepressants (TCA), an opioid, are commonly used for moderate to severe HN patients, their efficacy varies among individuals (Hempenstall et al., 2005; Johnson and McElhaney, 2009; Cohen, 2013; Johnson and Rice, 2014; Huffman et al., 2017; Parsons et al., 2018). In addition, some of these agents are not well-tolerated in HZ patients with multiple co-morbidities (e.g., renal or hepatic impairment), especially for elder patients (Schmader et al., 2010). Therefore, to reduce polypharmacy and provide treatment for patients who have failed medication, spinal neuromodulation techniques have been used to treat PHN over the past few decades (Lin et al., 2019; Aggarwal et al., 2020).

In recent years, with the development of technology and the economy in China, Chinese pain physicians have recruited temporary spinal cord stimulation (tSCS) to relieve severe pain syndrome in HN patients (Harke et al., 2002; Iseki et al., 2009; Moriyama, 2009; Yanamoto and Murakawa, 2012; Kurklinsky et al., 2018). A large number of patients, especially those with acute and subacute HN, experienced significant pain relief after tSCS treatment, improved quality of life, and greatly reduced the incidence of PHN (Dong et al., 2017; Han et al., 2020; Huang et al., 2020; Liu et al., 2020; Wan and Song, 2021). However, these studies were serial reports, retrospective analyses, or single-center prospective studies, and the level of evidence was not high enough. This makes tSCS treatment difficult to incorporate into various guidelines. It's critical that in clinical practice the application of tSCS treatment is based on medical evidence and, in cases where deficiency in the evidence exists, the clinical practice is improved by expert consensus opinion in the field. This is the rationale of the present study to demonstrate the existing opinions of pain physicians from the Chinese Pain Expert Working Group of the Chinese Neuromodulation Society (CNS) and the Chinese Medical Doctor Association (CMDA) on the clinical use of tSCS in HN treatment. To express this clearly, we use short-term spinal cord stimulation (st-SCS) instead of tSCS in the following context.

## Methods

Surveys to evaluate the analgesic efficacy and safety of st-SCS in HN syndrome were conducted in October 2021 by the Chinese Pain Expert Working Group of the CNS and CMDA. Each participant provided written informed consent to participate in this study. The contents of the questionnaire include basic information about each physician (training, hospital level, work experience, procedure numbers, etc.), efficacy, indications, and contraindications of st-SCS, operation conditions, and preoperative preparation of st-SCS, and the prospect of the st-SCS procedure. The survey was piloted by three senior experts for effectiveness, comprehensibility, and acceptability, with minor modifications based on the online survey, allowing respondents to rate whether all items should be included. Finally, the questionnaire was generated through an online survey of physicians in all tertiary public hospitals performing the procedure. Categorical data are presented as percentages and the number of cases. Data were analyzed and tabulated using Microsoft Excel 2019 (Microsoft Corporation, Redmond, WA, USA).

## Results

**Figure 1:**
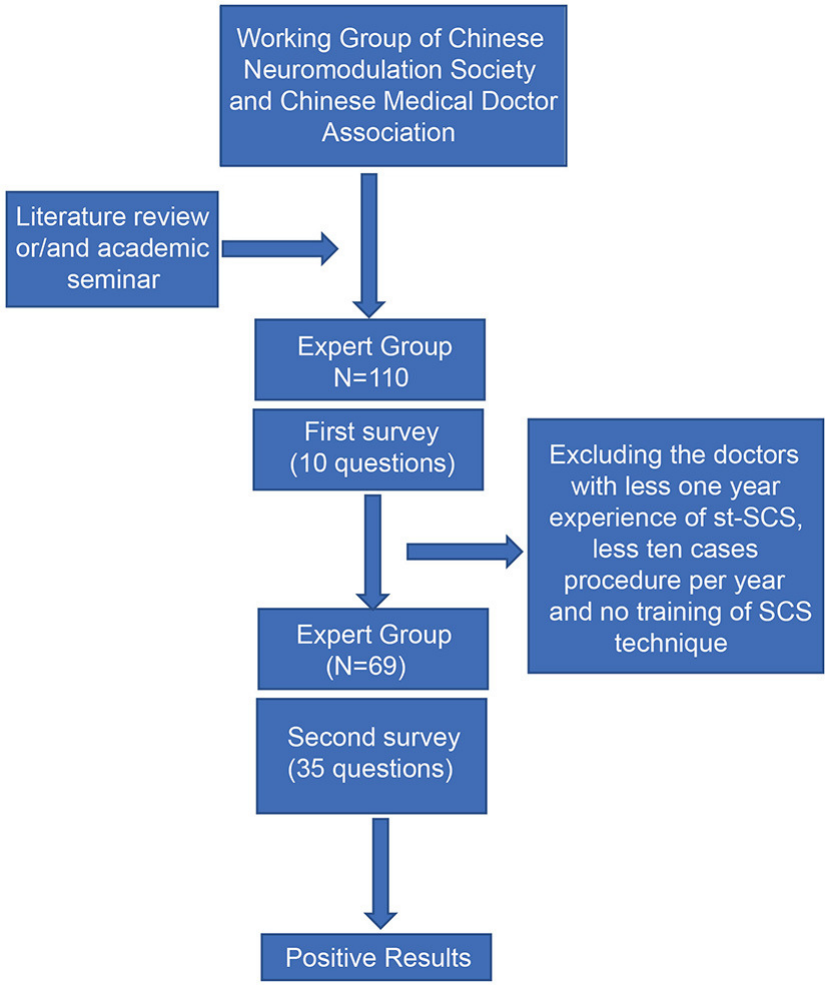
Schematic diagram of the consensus process.

Initially, 110 physicians from 91 hospitals who had performed st-SCS procedures participated in the survey, and 110 valid questionnaires were obtained. The st-SCS technique has almost been practiced by pain physicians, and the data from 110 questionnaires represent the overall situation of st-SCS in the treatment of HN in China. Based on these 110 questionnaires, an estimated 5,000–10,000 electrical stimulations are performed annually across the country. To obtain truly valuable information on st-SCS for HN, data from questionnaires for three conditions were excluded. The questionnaires were from physicians with <1 year of experience in st-SCS surgery, <10 procedures per year, and no SCS-specific standard training for the program. Finally, the results of 69 valid questionnaires obtained from senior pain physicians were incorporated with more valuable information on the efficacy, indications, and contraindications of st-SCS for the treatment of HN (Figure 1). Among the 69 pain physicians, 21 (30.43%) worked in SCS for 1–3 years, 17 (24.64%) worked for 3–5 years, and 31 (44.93%) worked for more than 5 years. Among them, 42 (60.87%) physicians performed 10–50 cases of st-SCS per year, 16 (23.19%) physicians performed 50–100 cases, and 11 (15.94%) physicians performed more than 100 cases per year (Table 1).

**Table 1:** General information of the pain physician who participated in this study.

	**Frequency (*n*)**	**Percentage**
**Hospital level**		
Tertiary hospital	68	98.54%
Secondary hospital	1	1.45%
First-class hospital	0	0
**Hospital level**		
Provincial	35	50.72%
Municipal	34	49.28%
County-level	0	0
**Job title**		
Chief physician	33	47.83%
Deputy chief physician	27	39.13%
Attending physician	7	10.14%
Physician	2	2.90%
**Department**		
Pain medicine	67	97.10%
Dermatology	0	0.00%
Neurosurgery	2	2.90%
Neurology	0	0.00%
Recovery unit	0	0.00%
Anesthesiology	0	0.00%
**HN outpatient cases**		
≤ 10	7	10.14%
10–30	13	18.84%
30–50	16	23.19%
50–100	20	28.99%
≥100	13	18.84%
**HN hospitalizations**		
≤ 10	1	1.45%
10–30	6	8.70%
30–50	5	7.25%
50–100	15	21.74%
≥100	42	80.87%
10~50	42	60.87%
50~100	16	23.19%
≥100	11	15.94%
**Carrying out st-SCS therapy for HN**		
1–3 yr	21	30.43%
3–5 yr	17	24.64%
≥5 yr	31	44.93%
**Have professional training**		
Continuing study for more than 3 months (in a hospital to develop this technology)	26	19.70%
Participated in physical training courses or observed	43	32.58%
Trained or observed this type of surgery abroad	27	20.45%
This type of surgery has been performed under the guidance of experts from outside of the hospital	36	27.27%

### Efficacy and safety of st-SCS in the treatment of HN

**Figure 2:**
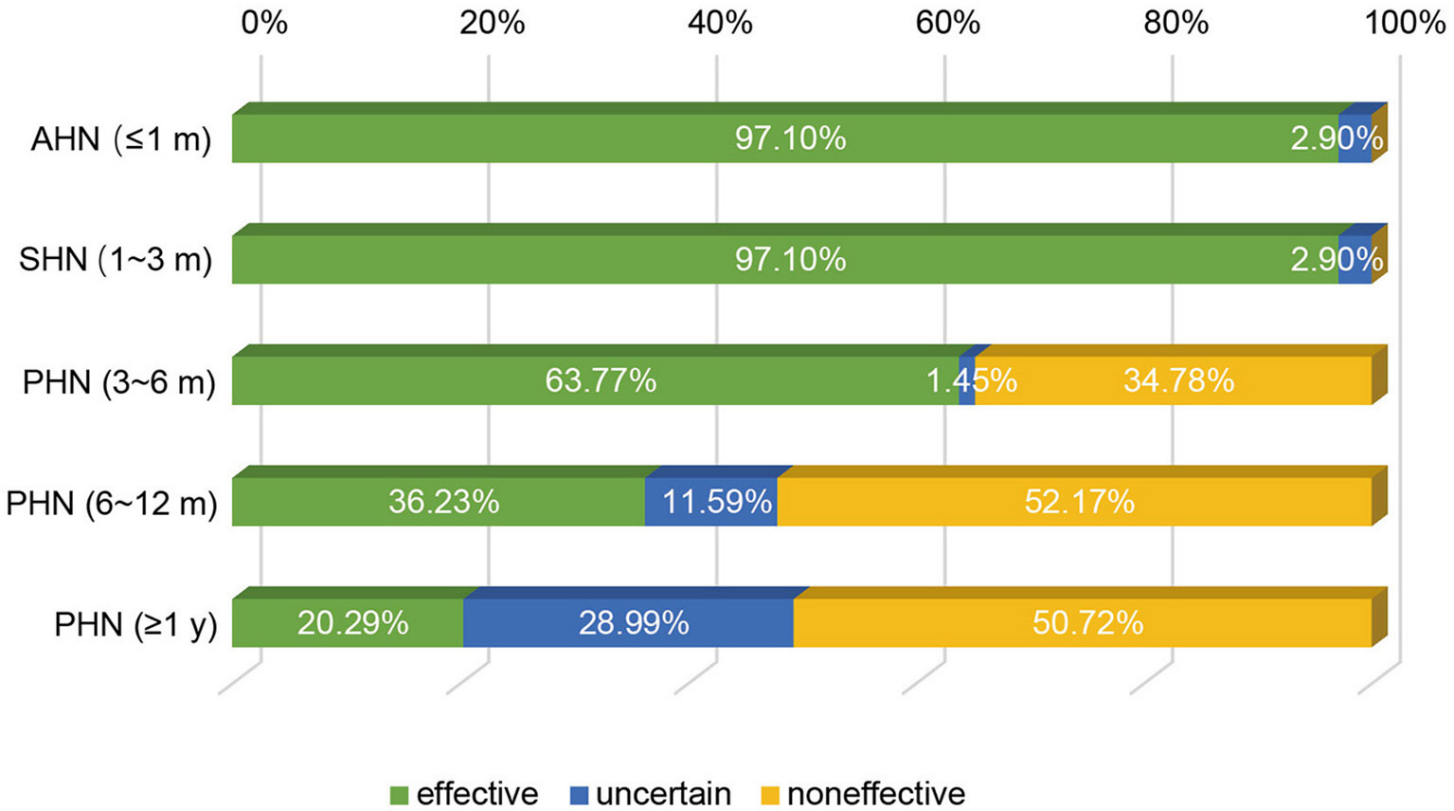
Efficacy of st-SCS on HN treatment. 67/69 (97.10%) experts agreed that HN patients achieved good curative effect within 3 months (patient satisfaction rate ≥50%). Early PHN patients (3–6 months) had the same curative effect (44/69, 63.77%). More than half of the experts agreed that st-SCS is ineffective in PHN patients with more than 6 months of history.

**Figure 3:**
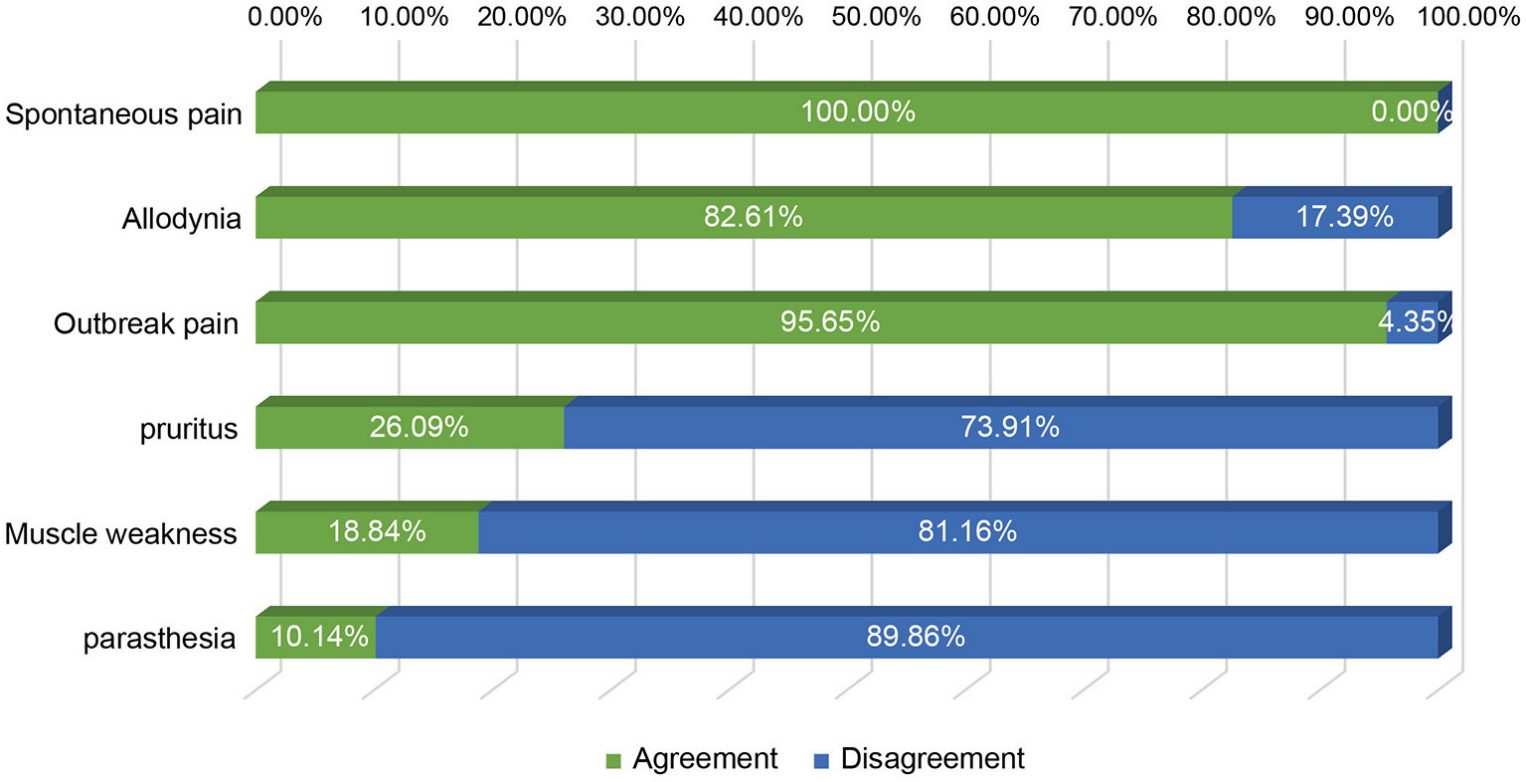
Improvement of HN symptoms by st-SCS. All experts agreed that treatment with st-SCS provided relief from spontaneous pain (100%), outbreak pain (95.65%), and allodynia (82.61%). Conversely, the more common disagreements were that the symptoms of paresthesia (89.86%), muscle weakness (81.16%), and pruritus (73.91%) could be improved by st-SCS.

**Figure 4:**
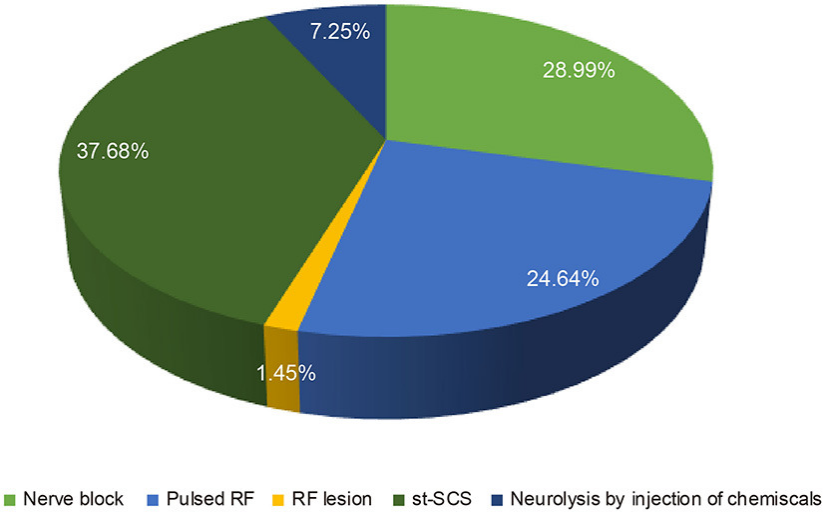
Comparison of the safety of different approaches for HN treatment. More physicians (26/76, 37.68%) choose st-SCS as the safety procedure, decrease by degree, nerve block (20/76, 28.99%), nerve pulsed radiofrequency (17/76, 24.64%), neurolysis with an injection of chemicals (5/76, 7.25%) and nerve radiofrequency lesion (1/76, 1.45%).

It is known that the efficacy of st-SCS in HN patients is related to the stage of HN. The results showed that it is generally believed (97.10%) that HN patients with <3 months can obtain a good curative effect (patient satisfaction rate ≥50%). Less agreement was achieved for over 1 year of PHN patients with good efficacy of st-SCS (Figure 2). Among the symptoms of HN, a large number of experts agreed that st-SCS treatment can relieve spontaneous pain (100%), outbreak pain (95.65%), and allodynia (82.61%). On the contrary, the more common divergence was that the symptoms of paresthesia (89.86%), muscle weakness (81.16%), and pruritus (73.91%) could be improved by st-SCS (Figure 3). Among the five surgeries used to treat HN in the clinic, doctors (26/76, 37.68%) preferred to choose st-SCS as the safest surgery, while nerve block (20/76, 28.99%), nerve pulse radiofrequency (17/76, 24.64%), chemically injected neurolysis (5/76, 7.25%), and nerve radiofrequency injury (1/76, 1.45%; Figure 4).

### Indications and contraindications for st-SCS therapy

**Figure 5:**
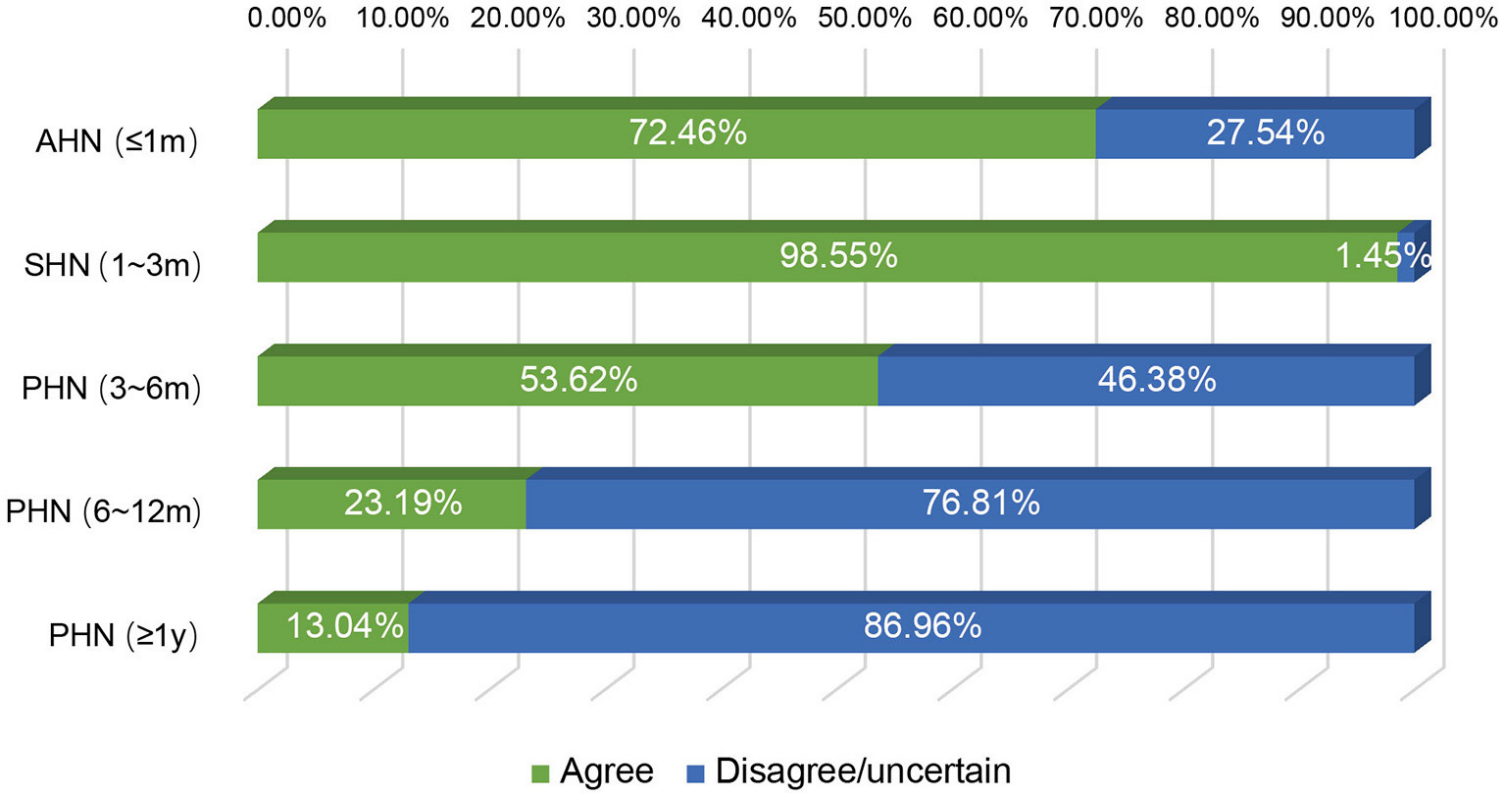
The indication of st-SCS for HN. Almost all (68/69, 98.55%) experts agreed that the st-SCS can be used in SHN patients, there was a common agreement (72.46%) that AHN patients are an indication of st-SCS and more than half agreement (53.62%) that st-SCS may be fit for early PHN (3–6 months). However, the majority (76.81 and 86.96%) of experts disagreed that PHN patients with more than 6 months and 1-year history should be considered as an indication of st-SCS.

**Figure 6:**
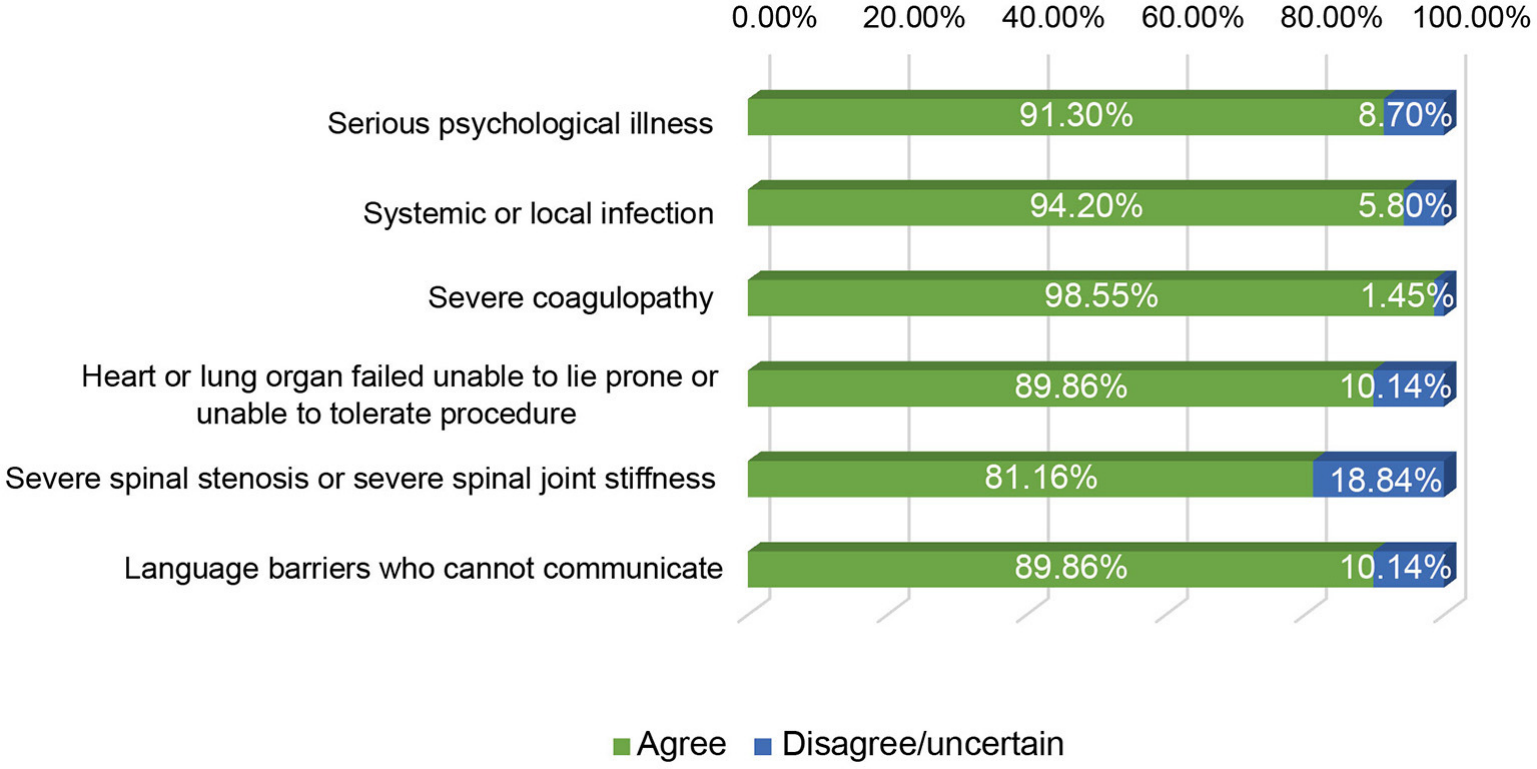
The contraindication of st-SCS for HN. More than 80% of experts agreed that the HN patients were contraindication for the procedure of st-SCS, who have the following situations of serious psychological illness, systemic or local infection, severe coagulopathy, heart, or lung organ failed unable to lie prone, or unable to tolerate the procedure, severe spinal stenosis, or severe spinal joint stiffness, language barriers who cannot communicate.

**Figure 7:**
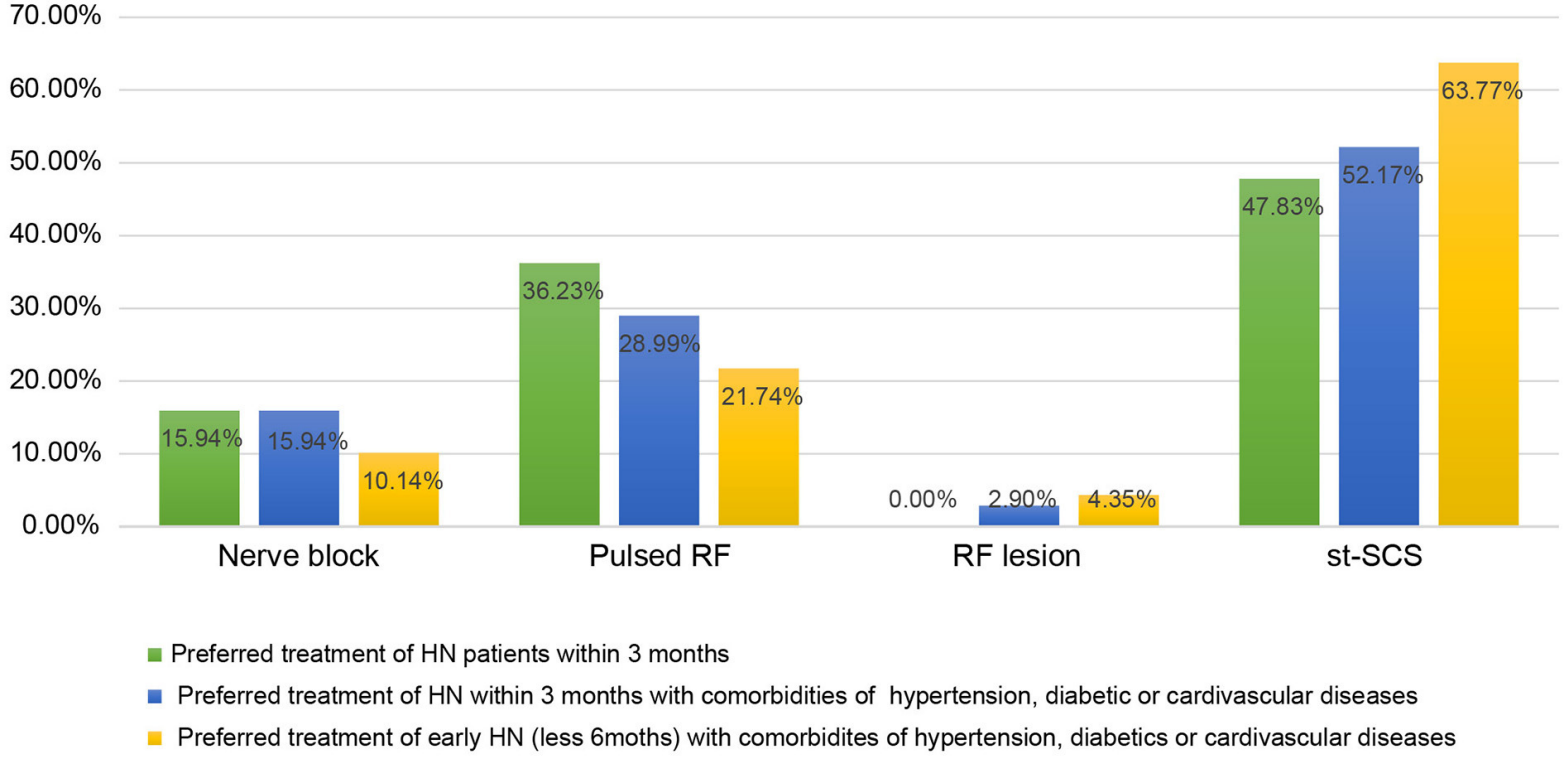
The preferred approaches for HN treatment among the techniques of nerve block, nerve pulsed RF, nerve radiofrequency lesion, and st-SCS. More experts agreed that st-SCS was the preferred treatment for HN within 3 months, especially for HN or early PHN patients with comorbidities of hypertension, diabetes, or cardiovascular disease, compared with nerve pulsed RF, nerve block, or nerve radiofrequency lesion.

We next investigated the indications and contraindications for st-SCS therapy. The results demonstrated that almost all (98.55%) physicians agreed that st-SCS can be used for SHN patients, there was a common agreement (72.46%) that AHN patients were an indication for st-SCS, and more than half (53.62%) agreed that st-SCS-SCS might be suitable for early PHN (3–6 m). However, the majority (76.81 and 86.96%) of experts disagreed that PHN patients with a history of more than 6 months and 1 year should be considered an indication for st-SCS (Figure 5). According to the results of this survey, a highly consistent consensus was achieved that a large majority (more than 80%) of experts agreed that HN patients are not suitable for the procedure of st-SCS, who have the following situations of serious psychological illness, systemic or local infection, severe coagulopathy, heart, or lung organ failed unable to lie prone or unable to tolerate the procedure, severe spinal stenosis or severe spinal joint stiffness, language barriers who cannot communicate (Figure 6). In addition, 33/69 (47.83%) of experts agreed that st-SCS was the preferred treatment for HN within 3 months, while 25/69 (36.23%) physicians preferred nerve pulsed RF, 11/69 (15.94%) physicians prefer blocked nerves. Choice of preferred st-SCS increased to 36/69 (52.17%) if HN patients had one or more comorbidities, such as hypertension, diabetes, or cardiovascular disease, and continued to increase to 44/69 (63.77%) for early PHN in patients with comorbidities (Figure 7).

### General information about the procedure of st-SCS

The most common agreement (92.75%) of not using st-SCS for HN treatment was because of the expansiveness. Before the procedure of st-SCS, spine examination of MRI was considered to be necessary for the majority (52/69, 78.26%) agreement, while 14.49% of them (10/69) have a contrary opinion and another five experts with an uncertain opinion. There was a consensus (53/69, 79.71%) was achieved that more than half of HN patients had experienced nerve block or nerve pulsed RF. A similarly large number of experts 57/69 (82.61%) agreed that an 80% paresthesia coverage should be achieved at the test stimulation and 57/69 (82.61%) agreed that the treatment of st-SCS needs to be persistent for 1–2 weeks. However, there is no consensus on the need for antibiotics during st-SCS (Table 2).

**Table 2:** General information about the procedure of st-SCS.

**General information**	**Agreement (%)**	**Dis-agreement (%)**	**Uncertain (%)**
St-SCS is too expensive to use	92.75	6.25	0
≥80% paresthesia coverage of the pain area should be achieved with the test stimulation	88.41	11.59	0
Treatment of st-SCS needs to be persistent for 1–2 weeks	82.61	17.39	0
Spine examination of MRI was necessary before st-SCS	78.26	14.49	7.24%
Antibiotics are necessary to be used during the procedure of st-SCS	52.17	30.43	17.39%

### Prospects of st-SCS for the treatment of HN

Regarding the prospects, a common consensus was reached on all statements regarding future research projects (Table 3). The procedure of st-SCS must be standardized (73.91% agreement) as well as the clinical application (e.g., indications, patient selection, and simulation time). Even in areas with allegedly good clinical data, such as treating HN within 3 months, the experts believe that more basic science and clinical research are still needed.

**Table 3:** Prospects of st-SCS for the treatment of HN.

	**Agreement (%)**	**Dis-agreement (%)**
Randomized controlled trials (RCTs) are necessary	69.57	30.43
A multi-center study should be conducted	75.36	24.64
Basic mechanism studies are necessary	68.12	31.88
Standardized training for pain physicians is required	73.91	26.09
Consensus or guideline is useful	73.91	26.09

## Discussion

Although the real-world data on the incidence, economic burden, and associated risk factors of HZ and PHN are still lacking in China (Sun et al., 2021), a cross-sectional study shows that the prevalence of HZ in China has been estimated to be 7.7%, and 29.8% of which develop to PHN (Yang et al., 2019), which also imposes a heavy burden on patients and society, as the HZ vaccination program in China is limited and the HZ vaccine coverage is far from optimal. Although early interventional treatment can provide fast and complete pain relief for HN and potentially reduce PHN incidence, it is known that multiple comorbidities suffered by elderly patients increase the risk of intervention complications. Furthermore, these interventions of spinal nerve, epidural, or sympathetic nerve block, and radiofrequency often fail to achieve long-term pain relief (Kumar et al., 2004; van Wijck et al., 2006; Moriyama, 2009; Dworkin et al., 2013; Gan et al., 2013; Kim et al., 2017). To ensure the safety of elderly HN patients, improve efficacy, and reduce complications, more and more Chinese pain physicians are interested in electrical stimulation therapy, especially st-SCS treatment (Huang et al., 2020). Thousands of early-stage HN patients have achieved very satisfactory treatment and accepted it as an excellent fast and effective alternative to medication with side effects (Huang et al., 2020; Liu et al., 2020). This is an expanded indication and application of neuromodulation that brings the promise of meeting the needs of patients and physicians. Our findings suggest that the application of st-SCS for HN treatment remains widely debated among pain physicians and neurosurgeons across the country.

### Indications and contraindications for st-SCS therapy

The greatest consensus exists in the field of indications or contraindications for st-SCS in the treatment of HN. Current literature suggests st-SCS for the treatment of AHN, SHN, and early PHN (<6 months; Yanamoto and Murakawa, 2012; Dong et al., 2017; Kurklinsky et al., 2018; Huang et al., 2020). Some experts agree that st-SCS also improves symptoms in PHN patients with a history of more than 6 months or even 1 year. However, there are still no large-scale studies to confirm this. In the present study, although most experts (76.81%) disagreed that PHN patients older than 6 months should be considered as an indication for st-SCS, many physicians would like to try st-SCS treatment first, and then permanent implantation of SCS might be possible if the symptoms of HN relapse after st-SCS treatment. More than 80% of agreements were achieved in HN patients, who have serious psychological illness, systemic or local infection, severe coagulopathy, heart or lung organ failed unable to lie prone or unable to tolerate the procedure, severe spinal stenosis, or severe spinal joint stiffness, language barriers who cannot communicate, are contraindications for the treatment of st-SCS.

### Safety of st-SCS therapy

SCS is generally considered a safe procedure, especially st-SCS, because it is characterized as minimally invasive and reversible, despite the permanent neurological deficit and severe spinal cord injury caused by epidural hematoma with epidural electrode implantation (Franzini et al., 2005; Santiago et al., 2005; Kloss et al., 2010; Smith et al., 2010; Boortz-Marx et al., 2012). However, no serious adverse events, such as cerebrospinal fluid leakage, or epidural hematoma, were observed during surgery and throughout the 1-year follow-up period, rather than complications such as some minor lead shifts and local infection at the puncture site (Huang et al., 2020). According to guidance from the Neuromodulation Appropriate Consensus Committee (NACC), SCS-related complications have been divided into patient-related, device-related, and technique or treatment-related complications, which are less threatening, although they can lead to treatment failure and device migration (Deer et al., 2014). To reduce the risks of implanting neurostimulation devices and improve outcomes, the NACC recommends that higher standards for training and quality of potential implanters are necessary (Deer et al., 2014). This is why this expert consensus does not include questionnaire data from physicians with <1 year of st-SCS experience, <10 procedures per year, or no training in standard SCS techniques. Although some interventional procedures such as nerve block and nerve pulse radiofrequency are also very commonly used for HN treatment in China, they can provide rapid and satisfactory pain relief for early PHN and may reduce the incidence of PHN (Dworkin et al., 2013). However, the multiple comorbidities experienced by older patients increase the risk of complications from the intervention. Furthermore, these interventions often fail to achieve long-term pain relief (van Wijck et al., 2006; Kim et al., 2017). Considering safety and efficacy, more physicians prefer st-SCS for HN patients, especially those with hypertension, diabetes, or cardiovascular disease.

## Limitation

The survey sought to obtain strong evidence to support the use of st-SCS in HN treatment, but its national character is a possible shortcoming of the survey. National differences in st-SCS medical management and reimbursement may influence conclusions. Furthermore, this consensus is not multidisciplinary and contains only the views of pain physicians. However, this can also be seen as an advantage as this is the only group actively conducting and monitoring st-SCS treatment. As this consensus was formed by a group of specialized pain physicians with substantial expertise in neuropathic pain and neuromodulation, both from a basic science perspective and from a clinical perspective.

## Recommendations

Based on the agreement of at least 70% of participating pain physicians, the expert consensus definition is as follows:

_ Patients with HN (including AHN and SHN) may experience significant benefit from st-SCS therapy within 3 months, and the use of st-SCS in HN (<3 months) therapy may be useful.
_ Electrode placement for st-SCS was considered adequate when patients reported 80% or greater paresthesia coverage of the painful area after test stimulation (Liu et al., 2020).
_ Treatment of st-SCS needs to be continued for 1–2 weeks (Han et al., 2020; Huang et al., 2020; Liu et al., 2020).
_ The procedure for st-SCS must be standardized.
_ Standardized training of SCS is required for pain physicians.

## Conclusion

The consensus is that HN within 3 months is a good indication for st-SCS therapy. However, more than 53.62% of experts agreed that early PHN (<6 months) may be a relative indication for st-SCS treatment. PHN patients with a history of more than 6 months can also try st-SCS therapy and then consider the next step based on the results, as this therapy is safe. Treatment of st-SCS needs to be continued for 1–2 weeks after electrode stimulation produces paresthesia covering ≥80% of the painful area. In addition, standardized training for pain physicians as well as for basic and clinical research is warranted.

## Future perspective

Although multiple kinds of the literature suggest that st-SCS reduces pain and improves the quality of life in HN patients <3 months old, the evidence from these studies is not solid enough. Therefore, it is still necessary to conduct clinical studies on the treatment of HN with st-SCS, such as randomized controlled trials (RCTs) and multicenter studies. Moreover, the mechanism of SCS in the treatment of HN also needs in-depth research. In addition, the existing wired electrical stimulation devices still cause a lot of inconvenience to patients. Therefore, non-invasive, and wireless electrical stimulation may be a new direction for future development.

## Data availability statement

The datasets presented in this study can be found in online repositories. The names of the repository/repositories and accession number(s) can be found in the article/Supplementary material.

## Ethics statement

Ethical review and approval was not required for the study on human participants in accordance with the local legislation and institutional requirements. The patients/participants provided their written informed consent to participate in this study.

## Author contributions

LX, WS, and YJ were responsible for the concept and design of the study, performed data presentation, and writing of the manuscript. All authors were involved with the data analysis and interpretation. All authors contributed to the article and approved the submitted version.

## Funding

This work was supported by grants from Guangdong provincial high-level key clinical of pain medicine, the Sanming project of Shenzhen Municipal Health Commission (SZSM202103018), Shenzhen Municipal Science, Technology and Innovation Commission (No. JCYJ20210324112202006), and the Clinical Frontier Technology Program of the First Affiliated Hospital of Jinan University, China (No. JNU1AF-CFTP-2022-a01212).

## Conflict of interest

The authors declare that the research was conducted in the absence of any commercial or financial relationships that could be construed as a potential conflict of interest.

## Publisher's note

All claims expressed in this article are solely those of the authors and do not necessarily represent those of their affiliated organizations, or those of the publisher, the editors and the reviewers. Any product that may be evaluated in this article, or claim that may be made by its manufacturer, is not guaranteed or endorsed by the publisher.
