# Supplementary material for: Short-term spinal cord stimulation is an effective therapeutic approach for herpetic-related neuralgia—A Chinese nationwide expert consensus
Source: Front Aging Neurosci. 2022 Sep 20;14:939432. doi: 10.3389/fnagi.2022.939432 (PMC9530637; doi:10.3389/fnagi.2022.939432)
Supplement: Supplementary file 1 [file Data_Sheet_1.DOCX]

**Questionnaire of short-term electrical nerve stimulation in the treatment of herpetic-related neuralgia**

Dear Directors and Colleagues:

To make the clinical use of short-term spinal cord stimulation for the treatment of herpetic-related neuralgia more standardized, a questionnaire survey is now launched on some related issues of short-term spinal cord stimulation in the treatment of herpetic-related neuralgia. Experienced colleagues actively participate in providing an experience for the clinical development and promotion of this technology to better benefit patients.

In this survey, herpetic-related neuralgia (HN) included acute (A cute herpetic neuralgia, AHN) within one month or subacute herpetic neuralgia within 1-3 months (SHN) and postherpetic neuralgia (PHN) for three months or more. HN refers to neuralgia caused by acute and subacute herpetic neuralgia, i.e., medical history ≤ 3 months.

Thanks for your support!

Neuromodulation Professional Committee of the Chinese Medical Doctor Association

September 28, 2021

1. Basic information

hospital name:

Your hospital belongs to:

□tertiary hospital, □secondary hospital, □primary hospital

Hospital level: □provincial □city □county level

Title: □Chief Physician □Deputy Chief Physician □Attending □Physician Physician

Department: □Pain Medicine □Dermatology □Neurosurgery □Neurology □Rehabilitation Department □□Others:

Fill in the phone number: Mail:

2. Questionnaire

1. How many patients with herpetic-related neuralgia (HN) does your outpatient department receive monthly?

A. ≤10_

B. 10~30

C. 30~50

D. 50~100

E. ≥100

1. How many inpatients with HN have your department treated each year?

A. ≤10_

B. 10~30

C. 30~50

D. 50~100

E. ≥100

1. How many nerve segments are usually involved in the patients you have seen with herpes zoster?

A. 1_

B. 2_

C. 3_

D. >3

E. The range of herpes is different in patients, and the invasion of nerve segments is different. It may be one or more than one.

1. How long has your department carried out the short-term electrical nerve stimulation treatment HN?

A. <1 year

B. 1~3 years

C. 3~5 years

D. >5 years

1. Have you received professional training before starting short-term electrical nerve stimulation therapy for HN (multiple choices)?

A. Continuing study for more than 3 months (study at the hospital that has already carried out this technology)

B. Participated in physical training courses or observed spinal cord electrical stimulation

C. Have trained or observed such operations abroad

D. Have performed such operations under the guidance of external experts

E. No training at all, based on experience or just listening to relevant lectures to carry out this technology independently

1. Does your department carry out short-term electrical nerve stimulation treatment for HN every year (the year with the most cases in the past 1-3 years)?

A. <10

B. 10 ~ 50

C. 50 ~ 100

D. >100

1. Which phase of the patient did you treat HN with short-term electrical nerve stimulation and achieved satisfactory results (patient satisfaction rate ≥ 50 %) (multiple choices):

1) AHN patients (<1 month);

2) SHN patients in subacute stage (1-3 months);

3) PHN patients with a medical history of 3 to 6 months;

4) PHN patients with a history of 6 to 12 months;

5) PHN patients with a history of 1 to 3 years;

6) PHN patients with medical history > 3 years;

1. Which type of patients do you think has the best effect in the treatment of HN with short-duration electrical nerve stimulation:

1) AHN patients (<1 month);

2) SHN patients in subacute stage (1-3 months);

3) PHN patients with a medical history of 3 to 6 months;

4) PHN patients with a history of 6 to 12 months;

5) PHN patients with a history of more than 1 year;

1. Based on your experience, what do you think of the efficacy of short-term electrical nerve stimulation for HN:

1) Acute phase HN (<1 month): A Valid; B Invalid; C Not sure;

2) Subacute stage HN (January to March): A Effective; B Ineffective; C Uncertain;

3) Medical history 3~ 6 months: A Valid; B Invalid; C Not sure;

4) Medical history from June to December: A Valid; B Invalid; C Not sure;

5) Medical history >1 year: A Valid; B Invalid; C Not sure;

1. In your experience, what symptoms can be improved in HN patients with short-term electrical nerve stimulation therapy? Can multiple choice

A. Spontaneous pain

B. allodynia

C. burst pain

D. itching

E. Decreased muscle strength

F. numbness

1. What do you think is the possible mechanism of short-duration electrical nerve stimulation in the treatment of HN (multiple choices):

A. Gate Theory

B. Regulation of sympathetic nervous system function

C. Anti -injury effect

D. Inhibit peripheral or central sensitization

E. The mechanism is unclear

1. According to your opinion, what are the better indications for short-term electrical nerve stimulation in the treatment of HN (multiple choices):

A. Acute phase H Z (<1 month)

B. Subacute HZ (January to March)

C. March to June

D. June to December

E. ＞1 year

1. Do you think what should be contraindicated in the treatment of HN with short-term electrical nerve stimulation (multiple choices):
2. serious mental illness;
3. In the presence of unhealed infection, severe infection at the puncture site, and (or) systemic;

C. Patients with severe coagulation dysfunction;

D. Patients with heart, lung, liver, kidney, and another important organ failure, unable to lie on their stomach or unable to tolerate surgery;

E. Severe spinal stenosis, severe spinal stiffness, or scoliosis;

F. Those who are unable to communicate due to language barriers;

G. Others (please note);

1. Minimally invasive treatment techniques for patients with HN, which one do you think is the safest (referring to the sum of the effects of manipulation or injection on the body)?

A. Nerve block therapy, excluding damaging drugs

B. Nerve Pulse Radio Frequency

C. Nerve radiofrequency damage

D. short-term electrical nerve stimulation

E. nerve trunk and other parts of the injection of chemical damage drug treatment

1. Which minimally invasive treatment would you consider first for a patient with HN within 1-3 months without comorbidities?

A. Nerve block therapy, excluding damaging drugs

B. Nerve Pulse Radio Frequency

C. Nerve radiofrequency damage

D. short-term electrical nerve stimulation

E. nerve trunk and other parts of the injection of chemical damage drug treatment

1. For HN patients with hypertension, diabetes, or cardiovascular and cerebrovascular diseases, which minimally invasive treatment method would you consider first?

A. Nerve block therapy, excluding damaging drugs

B. Nerve Pulse Radio Frequency

C. Nerve radiofrequency damage

D. Short-duration spinal cord stimulation

E. Nerve trunk and other parts of the injection of chemical damage drug treatment

1. For PHN patients with hypertension, diabetes, or cardiovascular and cerebrovascular diseases, which minimally invasive treatment method would you consider first?

A. Nerve block therapy, excluding damaging drugs

B. Nerve Pulse Radio Frequency

C. Nerve radiofrequency damage

D. short-term electrical nerve stimulation

E. nerve trunk and other parts of the injection of chemical damage drug treatment

1. Which minimally invasive approach would you consider first for a patient with PHN with a history of more than 6 months?

A. Nerve block therapy, excluding damaging drugs

B. Nerve Pulse Radio Frequency

C. Nerve radiofrequency damage

D. Short-term electrical nerve stimulation

E. Nerve trunk and other parts of the injection of chemical damage drug treatment

1. In addition to short-term electrical nerve stimulation, the minimally invasive treatment techniques for HN also carried out by your department include: (multiple choices)

A. All types of nerve blocks or nerve injections, excluding damaging drugs;

B. Nerve Pulse Radio Frequency;

C. Nerve radiofrequency damage;

D. Intradermal injection;

E. Nerve trunk and other parts of the injection of chemical damage drug treatment;

F. Other please specify

above minimally invasive techniques, the ones you use the most are:

A. Various types of nerve blocks or injections of nerve drugs;

B. Nerve Pulse Radio Frequency;

C. Nerve radiofrequency damage;

D. short-term electrical nerve stimulation;

E. intradermal injection;

F. other please specify;

1. Which short-term electrical nerve stimulation treatment would you prefer for a patient with HN of the head and face:
2. Supraorbital or infraorbital nerve stimulation
3. Foramen ovale or foramen ovale nerve trunk electrical stimulation;
4. Semilunar ganglion electrical stimulation
5. Spinal cord stimulation of the upper cervical segment
6. occipital nerve;
7. Which electrode implantation method would you prefer for HN patients in the upper cervical segment (posterior occipital or cervical plexus range):

A. Spinal cord stimulation;

B. Spinal nerve root electrical stimulation;

C. Dorsal root ganglion (DRG) electrical stimulation;

D. Peripheral nerve stimulation;

1. Which electrode implantation method would you prefer for HN patients with upper extremity involvement:

A. Spinal cord stimulation;

B. Spinal nerve root electrical stimulation;

C. Dorsal root ganglion electrical stimulation;

D. Peripheral nerve stimulation;

1. For thoracic and abdominal HN patients, which electrode implantation method would you prefer:

A. Spinal cord stimulation;

B. Spinal nerve root electrical stimulation;

C. Dorsal root ganglion electrical stimulation;

D. Peripheral nerve stimulation;

1. Which electrode implantation method would you prefer for lower extremity HN patients:

A. Spinal cord stimulation;

B. Spinal nerve root electrical stimulation;

C. Dorsal root ganglion electrical stimulation;

D. Peripheral nerve stimulation;

1. Which electrode implantation method would you prefer for sacrococcygeal HN patients:

A. Spinal cord stimulation;

B. Spinal nerve root electrical stimulation;

C. Dorsal root ganglion electrical stimulation;

D. Peripheral nerve stimulation;

1. Electrode implantation in trunk HN patients, under what conditions did you perform it?

A. DSA Room;

B. The sterile room of the C-arm machine with real-time fluoroscopy;

C. CT room;

D. Under the guidance of ultrasound in the treatment room;

1. Who do you think should be responsible for the postoperative programming of HN with short-term electrical nerve stimulation?

A. Team of doctors;

B. Factory engineer;

C. Family members or the patient himself;

D. Completed by the manufacturer under the guidance of the doctor;

1. Will you perform an MRI of the corresponding segment before using short-duration electrical nerve stimulation to treat HN?

A. Regularly do;

B. It depends on the situation;

C. Do not do it routinely;

1. Have your patients received nerve block or pulsed radiofrequency in addition to drug therapy before short-duration electrical nerve stimulation for HN?

A. 100 % _ _

B. 75 % -100 % _ _ _

C. 50 % -75 % _ _

D. 25 % -50 % _

E. ＜25 % _

F. 0 % _

1. During the electrode implantation test, when the tingling sensation needs to cover the pain area, do you think the electrode is in place and no further adjustment is required?

A. ＞50%

B. >80%

C. 100%

D. From personal experience, the coverage area is not important

E. In other cases, please note

1. What do you think is the best duration of short-term electrical nerve stimulation treatment?

A. 1 week

B. 2 weeks

C. >2 weeks

D. The short medical history can be 1 week, and the long medical history can be more than 2 weeks

E. other

1. Based on your experience/analysis, what are the common adverse effects of short-term electrical nerve stimulation? 【Multiple Choice】

A. Nerve damage

B. Vascular injury and bleeding, hematoma (including epidural hematoma)

C. Wound infection

D. Cerebrospinal fluid leakage or even low intracranial pressure headache

E. Electrode displacement even affects the curative effect

F. The tingling is sometimes absent, strong and weak

G. The patient does not adapt to the tingling sensation

H. Other Please note

**Sort by incidence:**

1. Do you think antibiotics are necessary for short-term electrical nerve stimulation treatment? When do you need to use it?

A. not required

B. Preoperative use

C. Intraoperative use

D. Postoperative use

E. It must be used before surgery, and it depends on the situation after surgery

F. Look at the situation before surgery, and use it after surgery

G. It will be all right

1. Do you think that placing two electrodes can improve the efficacy of short-term electrical nerve stimulation in the treatment of HN? If placing two electrodes, such as placing:

A. One electrode is enough, no need for two electrodes

B. Place two dorsal column electrodes

C. a dorsal column electrode, Another electrode is placed next to the pedicle to stimulate the nerve root

D. One dorsal column electrode and the other electrode are placed in the intervertebral foramen to stimulate the DRG

E. One dorsal column electrode and another electrode are placed peripherally to stimulate the nerve trunk or peripheral nerves

F. One electrode is placed next to the pedicle to stimulate the nerve root, and the other electrode is placed peripherally to stimulate the nerve trunk or peripheral nerves

1. During short-term electrical nerve stimulation therapy, to avoid electrode displacement, which statements about the post-operative patient position requirements are correct? (Multiple choice)

A. Peripheral nerve electrical stimulation does not require post-operative patient postural activity;

B. When the electrode is located in the cervical segment, there are strict requirements for the patient's position after surgery;

C. When the electrode is located in the thoracic segment, there are strict requirements for the patient's position after surgery;

D. When the electrode is located in the lumbar segment, there are strict requirements for the patient's position after surgery;

E. When the electrode is located in the intervertebral foramen for DRG electrical stimulation, there are strict requirements on the patient's position after surgery;

1. What extent do you think short-term electrical nerve stimulation is effective?

A. Pain or sleep improved by 30 % or more

B. 50% and above

C. 80%

D. Others, please note

37. For HN patients who are taking analgesics, during short-term electrical nerve stimulation therapy, your patient:

A. Stop analgesics before surgery

b. If the postoperative effect is good, stop using the analgesic, and if the effect is not satisfied, stop using the analgesic

c. Even if the postoperative effect is good, the analgesic drug needs to be tapered gradually

After 1-2 weeks of short-duration electrical nerve stimulation, what percentage of your HZN patients do you estimate still require oral analgesics? (_ ___% )

1. What do you think is the reason why short-term electrical nerve stimulation therapy for HN has not been widely carried out (multiple choices)?

A. The price is too expensive

B. Program control trouble

C. The operation is complicated

D. The mechanism is unknown

E. Poor efficacy

F. Others please note

1. The concept of using short-term electrical nerve stimulation to treat HN, please tick if you agree

A. is effective for herpes zoster-related neuralgia (including acute phase, subacute phase, and sequelae phase)

B. can significantly reduce the incidence of PHN in patients with acute and subacute herpes

C. Considering the safety and efficacy, it can be used as the first-choice minimally invasive treatment for patients with moderate and severe HN

D. is the first-choice treatment for PHN patients within one year in terms of safety and efficacy

E. Patients with PHN greater than 1 year or longer can also be considered for short-term electrical nerve stimulation therapy, and if there is a recurrence, permanent spinal cord stimulation can be considered

F. Compared with pulsed radiofrequency therapy, the curative effect is better and the maintenance time is longer

H. Can replace nerve block therapy

1. Application of short-term electrical nerve stimulation in the treatment of HN:
2. First choice for the treatment of patients with moderate-to-severe HN

B. Can be used as the preferred method for the treatment of patients with moderate to severe HN

C. Alternative treatment methods when other minimally invasive treatments are ineffective

D. Not considered at all

1. What do you think should be prioritized in the future for the treatment of HN with short-term electrical nerve stimulation

A. Mechanism research

B. Randomized Controlled Studies

C. Multicenter Study

D. Convening industry experts to write expert consensus or guidelines, and conduct standardized training for practitioners
